# Spatio-temporal dynamics and aetiology of proliferative leg skin lesions in wild British finches

**DOI:** 10.1038/s41598-018-32255-y

**Published:** 2018-10-10

**Authors:** Becki Lawson, Robert A. Robinson, Julia Rodriguez-Ramos Fernandez, Shinto K. John, Laura Benitez, Conny Tolf, Kate Risely, Mike P. Toms, Andrew A. Cunningham, Richard A. J. Williams

**Affiliations:** 1 Institute of Zoology, Zoological Society of London, Regent’s Park, London, NW1 4RY UK; 2 British Trust for Ornithology, The Nunnery, Thetford, Norfolk, IP24 2PU UK; 3 IDEXX Laboratories Limited, Grange House, Sandbeck Way, Wetherby, West Yorkshire LS22 7DN UK; 4 Departamento de Genética, Fisiología y Microbiología, Facultad de Ciencias Biológicas, Universidad Complutense de Madrid, E-28040 Madrid, Spain; 5 Zoonotic Ecology and Epidemiology, EEMiS, Linnaeus University, Kalmar, 391 82 Sweden; 6 Departamento de Biodiversidad, Ecología y Evolución, Facultad de Ciencias Biológicas, Universidad Complutense de Madrid, E-28040 Madrid, Spain

## Abstract

Proliferative leg skin lesions have been described in wild finches in Europe although there have been no large-scale studies of their aetiology or epizootiology to date. Firstly, disease surveillance, utilising public reporting of observations of live wild finches was conducted in Great Britain (GB) and showed proliferative leg skin lesions in chaffinches (*Fringilla coelebs*) to be widespread. Seasonal variation was observed, with a peak during the winter months. Secondly, pathological investigations were performed on a sample of 39 chaffinches, four bullfinches (*Pyrrhula pyrrhula*), one greenfinch (*Chloris chloris*) and one goldfinch (*Carduelis carduelis*) with proliferative leg skin lesions and detected *Cnemidocoptes* sp. mites in 91% (41/45) of affected finches and from all species examined. *Fringilla coelebs* papillomavirus (FcPV1) PCR was positive in 74% (23/31) of birds tested: a 394 base pair sequence was derived from 20 of these birds, from all examined species, with 100% identity to reference genomes. Both mites and FcPV1 DNA were detected in 71% (20/28) of birds tested for both pathogens. Histopathological examination of lesions did not discriminate the relative importance of mite or FcPV1 infection as their cause. Development of techniques to localise FcPV1 within lesions is required to elucidate the pathological significance of FcPV1 DNA detection.

## Introduction

The epidemiology and impact of diseases of wild birds that cause morbidity, but are not typically associated with mortality, are often poorly understood. Papillomatosis and cnemidocoptosis are each diseases characterised by proliferative leg skin lesions in British finches (family Fringillidae) which, although often seen, are infrequently reported as causing mortality. The chaffinch (*Fringilla coelebs*) is most frequently affected and lesions typically affect the non-feathered areas of the legs, feet and digits^1–3^. When severe, the skin lesions can interfere with locomotion, lead to digit distortion, loss and/or lameness and may predispose to entanglement or predation^1,3^ (BL *personal communication*).

Papillomaviruses are epitheliotropic, double-stranded DNA viruses that are generally considered host-specific^2,3^. *Fringilla coelebs* papillomavirus (FcPV1) is one of eight known to infect birds^4–8^. The small number of avian papillomavirus species is in contrast to the high number detected in mammals. Of known papillomaviruses, 344 of 353 are found in mammals; of which 180 are known only from humans (the Papillomavirus Episteme is available at https://pave.niaid.nih.gov/). It is unclear whether the relative paucity of papillomaviruses in non-mammalian taxa is real or due to sampling bias^9^.

Chaffinches with proliferative leg skin lesions associated with papillomavirus infection have been described from multiple countries in western, northern and central Europe, including the Czech Republic, Germany, Great Britain, Italy, Sweden and the Netherlands^6,10–14^. Papillomavirus from similar leg skin lesions has been reported less commonly in wild brambling (*Fringilla montifringilla*), wild bullfinch (*Pyrrhula pyrrhula*) and captive greenfinch (*Chloris chloris*)^2,6,15^. The macroscopic appearance of these proliferative leg skin lesions has been described generally as (squamous) papillomas and wart-like growths and more specifically as nodular or hyperplastic lesions with deeply fissured papillary growths (i.e. papilliferous)^2,3,14^. Microscopic examination has revealed a variety of abnormalities, including epidermal hyperplasia, papillary projections, hyperkeratosis, keratinocyte vacuolation, acanthosis, enlarged and homogenous epidermal nuclei with the appearance of intranuclear inclusion body formation^2,16,17^.

*Cnemidocoptes jamaicensis* (Family: Cnemidocoptidae) is a microscopic burrowing mite known to cause skin disease in numerous bird species, including ten species of European finch^18^. The macroscopic appearance of affected skin in wild birds ranges from grey/white diffuse scale, sometimes with a “powdery” or desiccated appearance, to severe hyperkeratosis with crusts and scab formation; the disease is known colloquially as ‘scaly leg’^1^. In addition to the detection of mites, microscopic examination has revealed a variety of abnormalities, many of which overlap with those observed in cases of viral papillomatosis, including epidermal hyperplasia, papillary projections, hyperkeratosis, keratinocyte vacuolation, acanthosis and variable evidence of inflammation with mixed cell infiltrate^1,18^.

Whilst it has been proposed that proliferative leg skin lesions in finches result from infection with either papillomavirus or cnemidocoptic mites^1–3^, co-detection of both of these pathogens has been documented only once previously: in a leg skin lesion from a single chaffinch^19^. Transmission of both FcPV1 and *Cnemidocoptes* sp. mites is believed to be via contact^1,2^.

Avian pox is a differential diagnosis for skin lesions in wild birds which may be confused with papillomatosis or cnemidocoptosis based on macroscopic appearance alone^2,16^. Microscopic examination can be used to confirm a diagnosis of avian pox based on the detection of pathognomonic intracytoplasmic inclusion (Bollinger) bodies^20^. Avian pox has been reported in various Fringillidae species in Europe, including the bullfinch, chaffinch, greenfinch, goldfinch (*Carduelis carduelis*), siskin (*Spinus spinus*) and common linnet (*Acanthis cannabina*)^20,21^.

There has been no large-scale investigation of the aetiology or epizootiology of proliferative leg skin lesions in wild finches to date. Here we, firstly, describe the occurrence of these lesions in finches across Great Britain (GB) in 2014 and 2015 and compare the findings from opportunistic and systematic surveillance. Secondly, we investigate the specific aetiology of the leg skin lesions in finches using samples collected from carcasses previously submitted for post-mortem examination (PME) from across England and Wales (April 2005–December 2015 inclusive), using a combination of parasitological, molecular and histopathological techniques. Finally, we consider whether the macroscopic appearance of the lesions is a reliable indicator of their aetiology.

## Methods

### Wild bird disease surveillance network

A national surveillance scheme to investigate the infectious and non-infectious diseases of British garden birds (initially the Garden Bird Health initiative, latterly incorporated into Garden Wildlife Health (GWH)) was launched in April 2005^22,23^. Reports of morbidity and mortality of garden birds were solicited from members of the public (opportunistic reports) and from participants of the British Trust for Ornithology’s Garden BirdWatch (GBW) scheme (systematic reports)^22,24^. From 2005 to 2013, reports were received via telephone or email. Since summer 2013, reports have been received online via the GWH website (www.gardenwildlifehealth.org). Each report detailed the numbers of each species affected, date, location and, when available, digital images of affected birds.

### Surveillance for chaffinches with leg lesions

Veterinarians reviewed all opportunistic disease incident reports from members of the public received on the GWH website in 2014 and 2015. Proliferative leg skin lesions affecting one or both legs of finches, consistent with cnemidocoptosis, papillomatosis, or co-infection with both (hereafter ‘leg lesions’), were identified on the basis of the observations and supporting images (where samples were not available to establish their aetiology and confirm the diagnosis). Avian pox was not considered as a differential diagnosis for the leg lesions since diagnostic testing performed in this study found no evidence of this aetiology (see results section). The geographic distribution of chaffinch leg lesion reports was summarised, with the total number stratified by the number of households by government office region (GOR), according to the most recent (2011) census data^25,26^, to account for variation in surveillance effort with human population density across GB. The seasonal distribution of these opportunistic incidents and the total number of affected chaffinches per site were summarised. We also considered western GB (the GORs North Scotland, North West England, South West England, Wales and the West Midlands) and eastern GB (the remainder) separately.

Systematic health surveillance was conducted by a subset of the network of circa 15,000 regular participants of GBW. Separate to the GWH web reporting system, this group reported whether they saw sick, diseased and/or dead individuals of any of the wild bird species on a weekly basis throughout the year. Using a web form (the ‘health tab’) in the GBW reporting system participants could report, among other options, “I did not see any disease/mortality incidents” or ‘Bird(s) with growths on leg’ in their garden during the previous week. Only regular online users of the ‘health tab’ (i.e. those who had recorded they had looked for the presence of any diseased individual in ≥40 weeks per year and who had reported observations of chaffinch in their gardens during the study period) were selected for analysis. Since the vast majority (96%) of opportunistic reports of finch leg lesions involved chaffinches (see below) and post-mortem examinations provided no common alternative differential diagnoses for such lesions in any other British wild bird species (BL *unpublished data*), we assumed the GBW reports of ‘birds with growth on legs’ represented cases of cnemidocoptosis, papillomatosis, or co-infection of both in chaffinches. Chaffinches were recorded at 97% of systematically-monitored sites from which ‘birds with growth on legs’ were reported. To make data handling manageable and to reduce problems induced by non-random spatial sampling (such as autocorrelation) we aggregated gardens by calendar week and GOR.

### Statistical analysis of temporal data

We estimated the mean relative reporting rates (the proportion of gardens reporting at least one chaffinch) each week in each of the 11 GOR from a generalised additive model (gam) with a smooth of calendar week number and an intercept for each GOR as fixed effects using the gam() function in R’s mgcv package^27,28^. We modelled the occurrence of lesions (as a proportion of those gardens which reported the presence of at least one chaffinch) as a function of calendar week (smoothed) and the number of chaffinches reported per garden using mgcv’s gamm() function. We incorporated GOR as a random effect, allowing the slope with chaffinch number also to vary regionally. For this analysis, we excluded three individual weeks in which anomalously high chaffinch numbers were reported, although including them did not alter our results (Supplementary Table S1). In both models, we used cyclic cubic regression splines^28^ to ensure that the fitted value for week 52 (last week of December) matched the fitted value for week 1 (the first week of January the following year) and specified a logit link and binomial error structure calculating the maximum likelihood estimate.

### Pathological and molecular examinations

When available, submission of freshly dead wild bird carcasses was encouraged for examination from both opportunistic and systematic reporters, April 2005–December 2015 inclusive. PMEs on birds from England and Wales were conducted at the Institute of Zoology following a standardised protocol. Body condition was assessed subjectively on the basis of pectoral muscle mass and body fat deposits. Liver, small intestinal contents and macroscopic lesions were routinely sampled and examined for the presence of pathogenic bacteria using a standardised protocol including *Salmonella*-selective enrichment media^29^. When present, oesophageal lesions were incubated at 30 °C in Trichomonas Media No. 2. (Oxoid, UK) and screened for motile trichomonads at 24, 48, 72 hrs and 5 days^30^. Cause of death (COD) categories (‘infectious disease’, ‘trauma’, ‘predation’, ‘other’, ‘undetermined’, or a combination) were assigned for each bird based on a review of all findings. The remaining carcass was archived at −20 °C following these investigations.

Digital photographs were taken of most birds with leg lesions that were examined *post mortem* to record the macroscopic lesion appearance. Diagnostic tests were performed to determine their aetiology; comprising parasitology, polymerase chain reaction (PCR) for papillomavirus and avian poxvirus and histopathology, although not all tests were performed in each case. First, a circa 5 mm^3^ sample of leg lesion was digested in warm 20% sodium hydroxide (NaOH) for circa 20–30 minutes and a crush preparation of this was examined using direct phase contrast light microscopy for evidence of *Cnemidocoptes* mites.

The remainder of each leg lesion was archived at −20°C and/or −80°C. DNA was extracted from thawed lesions using either the Biosprint 15 DNA Blood Kit (IOZ) or DNeasy Blood and Tissue Kit (UCM) (both Qiagen, UK) following the manufacturer’s instructions for purification of DNA from tissue. Multiple DNA extraction negative controls (reagents only and no tissue sample) were included in each batch to confirm the absence of cross-contamination during DNA extraction.

To explore whether apparently healthy birds form a reservoir of latent papillomavirus infection, a sample of digit and tarsometatarsus was collected from 40 chaffinches with no detected macroscopic skin abnormalities from the available archive. DNA was extracted from a pooled sample of the digit and tarsometatarsus from each of these chaffinches and screened for papillomavirus DNA using the same PCR protocol. These ‘negative control’ chaffinches were selected from 2011–2015 inclusive with widespread spatial distribution from across GB including all available COD categories.

Extracted DNA was tested using a multiplex PCR designed to detect papillomavirus and avian poxvirus, using the primer pair *BconPV* (forward 5′-TYCCWAAGGTSTCTGSAATCA-3′ and reverse 5′-CCRAAGCCAATATCKSACAT-3′), targeting the L1 major capsid protein and P4b1060 (forward 5′-GATGGCTGACGAGGAACAAAT-3′ and reverse 5′-TAGCCGGCATAAACATAACTCTTC-3′), targeting the P4b gene, to detect the pathogens respectively^31^. Each run included a plasmid positive control, PCR negative control consisting of molecular grade water and a DNA extraction negative control. The positive control contained simple PCR products of FcPV1 (sample MADE 21^31^) and Avipox (from coal tit *Periparus ater*) inserted into cloning vectors following manufacturer’s instructions (TOPO PCR cleaning kit; Invitrogen, Carlsbad CA, USA). All samples were re-tested in a simple PCR with the *BconPV* primers (specific for avian papillomavirus) to confirm the results for positive samples. Amplified DNA was visualised by loading 5 μl of PCR product in 1.5% agarose gels stained with Gel Red (Biotium, Heyward, CA, USA). Aliquots of positive samples were purified and sequenced using a Perkin-Elmer 2400 thermal cycler (ABI Prism Dye Terminator Cycle Sequencing Ready Reaction Kit; Applied Biosystems, Foster City, CA, USA). Bidirectional Sanger sequencing of products was conducted using an ABI Prism 3730 automated sequencer (ThermoFisher Scientific, Waltham, MA, USA). The nucleotide sequences obtained were analysed with Lasergene software (DNAStar, Madison, WI, USA). The nucleotide consensus sequences were compared with known avian and mammalian papillomavirus sequences available in GenBank by using the NCBI BLAST software (http://www.ncbi.nlm.nih.gov/blast/Blast.cgi).

Subgross examination (at x40 magnification) of intact skin on the legs of ‘negative control’ chaffinches, i.e. those with no macroscopic leg abnormalities, but that were PCR-positive for FcPV1 DNA, were examined using a Zeiss Stemi SV6 microscope (Carl Zeiss, Oberkochen, Germany), with a Motic Moticam 2500 lens (MoticEurope S.L.U., Barcelona, Spain) and visualized using their Image Plus 2.0 software.

Sections of leg lesion were fixed in neutral-buffered 10% formalin and processed for histopathological examination (both transverse and longitudinal orientations, where lesion size permitted) using routine methods with Haematoxylin and Eosin staining. Sections from all available cases were examined blind of other ancillary diagnostic results by a single veterinary pathologist (JRRF). The presence and severity of the following features of skin disease were described in each case, some of which reflect those previously described in finches with either papillomatosis, cnemidocoptosis or mixed infection (see above): hyperkeratosis, epidermal hyperplasia, intranuclear inclusion bodies, keratinocyte vacuolation, intercellular oedema, dermal inflammation, bacterial colonies, mites and epidermal papillary projections. *Cnemidocoptes* sp. mites were detected by either a NaOH digest or histopathological examination. Papillomavirus DNA was detected by PCR with product identity confirmed by sequencing. The histopathological findings were then reviewed in the knowledge of the other diagnostic test results, to evaluate if microscopic abnormalities can discriminate between infection with papillomavirus and *Cnemidocoptes* mites.

Finally, available macroscopic images were reviewed blind and described by the veterinarian who conducted the majority of the PMEs (BL). Lesions were scored by severity (mild, moderate, severe) according to their size, character and the extent of leg involvement and classified into one of three categories: 1. papilliferous lesions (as previously described in finches with papillomatosis^2^), 2. generalised scale and/or cornified proliferative lesions with crust formation (as previously described in finches with cnemidocoptosis^1^), or 3. mixed or intermediate appearance. Veterinary opinion was compared with the diagnostic test results to determine the reliability of aetiological diagnosis from visual examination of macroscopic lesions alone.

## Results

### Surveillance

Opportunistic reports of chaffinch leg lesions were received from 89 sites in 2014 and 80 sites in 2015: these comprised reports from all GORs in 2014 and all GORs except North East England in 2015. Over the same two year period, leg lesions in bullfinches were only reported from five sites and in goldfinch and greenfinch from one site each. A single affected finch species was observed at each site (i.e. chaffinch, bullfinch, goldfinch or greenfinch alone). Where photographs of affected finches were submitted, the range of macroscopic lesion appearance was similar to that observed in confirmed cases by PME.

**Figure 1:**
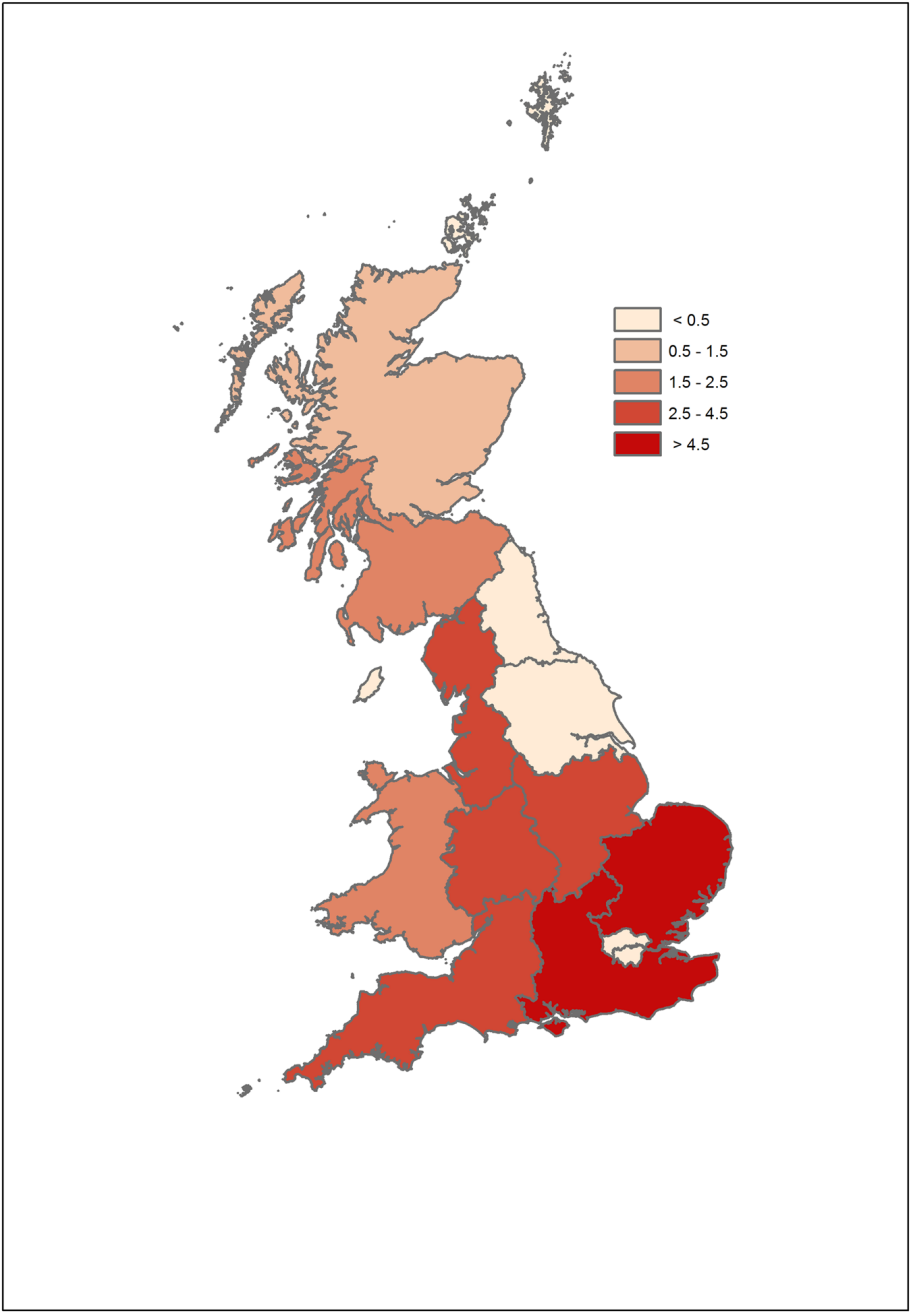
Regional occurrence of chaffinch leg lesion reports in 2014–2015 reported by opportunistic surveillance. Shading indicates the number of gardens reporting diseased individuals through the Garden Wildlife Health website (www.gardenwildlifehealth.org) per 100,000 gardens in the region. Map was created using ArcMap 10.0 (https://desktop.arcgis.com/en/arcmap/).

**Figure 2:**
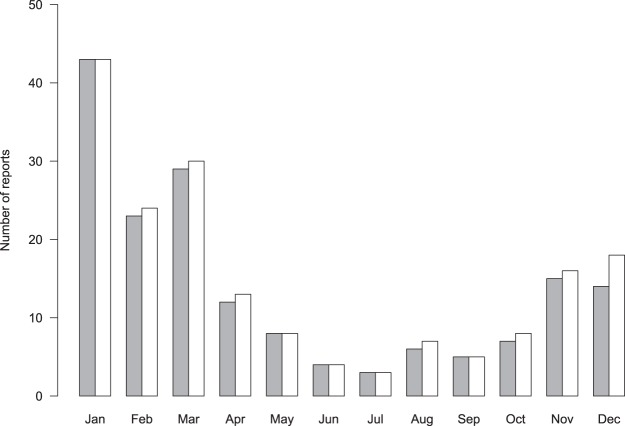
Seasonal occurrence of chaffinch leg lesion reports in 2014–2015. Bars indicate the total number of opportunistic reports to the Garden Wildlife Health website (www.gardenwildlifehealth.org) by month for the first month a report was received at each site per annum (filled bars) and for the total number of reports received for each month across sites (open bars).

There was little apparent difference in the spatio-temporal distribution of chaffinch leg lesions reports between 2014 and 2015 (Supplementary Fig. S1a,b); therefore combined data are presented (Fig. 1). Incident reports from the opportunistic scheme were widely distributed across GB, but most frequently from the East of England GOR. There was a clear seasonal peak, November to March (Fig. 2). Most sites (69%) reported one chaffinch with leg lesions (62/89 in 2014; 55/80 in 2015); the mean number of affected chaffinches per site was 1.9 (range 1–10) in 2014 and 1.7 (range 1–12) in 2015. Over the two-year study period, observations of systemic ill health (e.g. fluffed up plumage, lethargy) of chaffinches with leg lesions were reported from a minority of sites (4%, 7/169) and mortality of a single affected chaffinch was observed at 8% (13/169) of sites. No incidents with mortality of multiple birds with leg lesions were reported.

**Figure 3:**
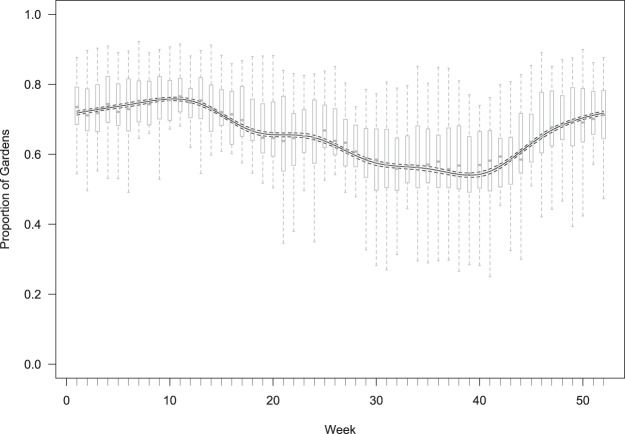
Seasonal occurrence of chaffinches in 2014–2015. The solid line indicates the mean proportion of gardens in the systematic survey reporting chaffinches each week and the dashed line the 95% confidence intervals about this line. The box and whisker plots summarise the mean percentage of gardens reporting chaffinches across the regions over both years.

Data collected systematically through the GWH scheme on the presence or absence of wild bird disease were available from approximately 3,000 regularly monitored sites across GB. Whilst the absolute number of monitored sites varied by GOR (reflecting variation in human population density), the number of weekly observations received during the 6-month breeding and 6-month non-breeding seasons remained consistent for each region, implying a comparable level of surveillance intensity throughout the year. The reporting rate of chaffinches in systematically monitored sites varied throughout the year, with numbers decreasing over the breeding season (April to September) and increasing through the non-breeding season to a peak in late March (Fig. 3). There was limited geographic variation in presence of chaffinches, with 65–70% of gardens in each region reporting chaffinches over the study period (Supplementary Fig. S2).

**Figure 4:**
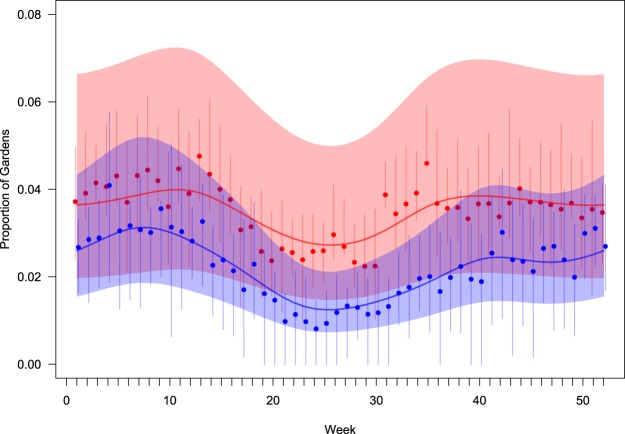
Occurrence of chaffinch leg lesions in eastern (blue) and western (red) Great Britain through the year. Solid lines indicate the relative mean occurrence (compared to the annual mean) through the year and shading indicates the 95% confidence region about the lines. Points indicate the mean and vertical bars the inter-quartile, reported occurrence in each region in each week.

**Figure 5:**
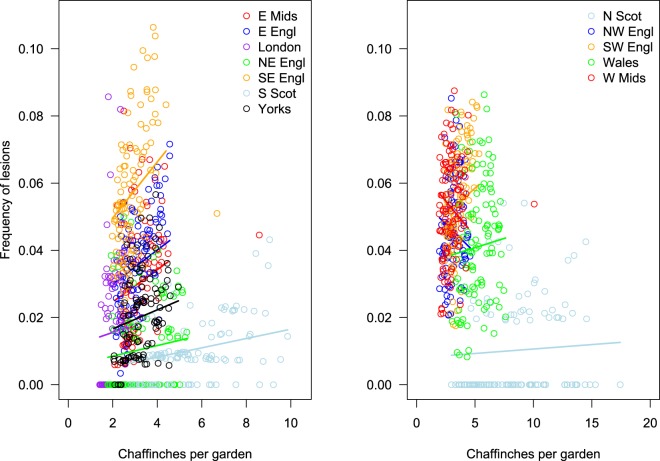
Proportion of gardens with birds with leg lesions in relation to the mean number of chaffinches reported. Each point represents a weekly mean for each region and points are coloured by region. Solid lines indicate the slopes for each region estimated from a GLMM with a random slope term; lines extend though the data included in the model (i.e. three anomalous points were excluded, see text).

Typically, each week 3–4% of participants who reported seeing chaffinches recorded at least one bird with leg lesions, with the highest frequency in winter and the lowest in mid-summer. The seasonal effect was greater in eastern, than in western GB (Table 1), although the increase in leg lesion reporting occurred earlier in western (late August/September) than in eastern regions (late September/October; Fig. 4). There was a consistent positive relationship between the frequency of leg lesion reports and the number of chaffinches recorded in gardens in the eastern regions of England (slopes β ~ 0.15), but this relationship was mixed with both positive and negative associations for the GORs in the western regions of England and in Wales (slopes β ~ 0) (Fig. 5; Supplementary Table S1).

**Table 1:** Summary statistics for models of seasonal variation in reporting of leg lesions in chaffinches from the systematic data.

Model	Estimated d.f.		ΔAIC
week	1		0
s(week)	6.26 (F = 23.3, p < 0.001)		−134.4
side + s(week)	6.22 (F = 22.7, p < 0.001)		−142.9
	East	West	
side + s(week): side	6.26 (F = 20.0, p < 0.001)	5.05 (F = 6.1, p < 0.001)	−148.3

Season is indicated by calendar week and models include either a linear (first model β = 0.0003 ± 0.0008, t = 0.4, p = 0.69) or a spline smooth, s(week), of season together with a fixed effect indicating side of country (East/West) either as an intercept (‘+’) or interaction (‘:’). The table gives the estimated degrees of freedom of the smooth (together with a test of whether this is different from 0) and the model AIC (expressed as the difference from the linear null model).

### Pathological and molecular examinations

A total of 1,066 finches of 10 species within the family Fringillidae were examined *post mortem*. Proliferative skin lesions affecting one or both legs were detected in 45 birds: 39 chaffinches (16%), 4 bullfinches (9%), 1 goldfinch (1%) and 1 greenfinch (0.2%) Table 2). Two affected chaffinches and one affected bullfinch were submitted from a single site in Shropshire, England, over a 5-month period and two affected chaffinches were submitted from a single site in Lancashire, England, over a 6-month period; all other affected birds were individual submissions per site. The majority of affected birds examined *post mortem* were found dead; the three finches submitted together from the Shropshire site and a chaffinch from a site in Wales were euthanased on welfare grounds due to the severity of their leg lesions. All birds examined were full-grown beyond their post-juvenile moult.

**Table 2:** Breakdown of finch species with leg lesions detected on post-mortem examination, April 2005–December 2015.

Species	Total no. of birds examined *post mortem*	Total no. of birds with leg lesions (%); from No. of sites	Sex of birds with leg lesions
Chaffinch *Fringilla coelebs*	250	39 (16%); 37	26 male; 11 female; 2 undetermined
Bullfinch *Pyrrhula pyrrhula*	45	4 (9%); 4	2 male; 1 female; 1 undetermined
Goldfinch *Carduelis carduelis*	91	1 (1%); 1	1 female
Greenfinch *Chloris chloris*	617	1 (0.2%); 1	1 undetermined
Siskin *Spinus spinus*	47	0	N/A
Lesser redpoll *Acanthis flammea*	4	0	N/A
Brambling *Fringilla montifringilla*	8	0	N/A
Hawfinch *Coccothrautes coccothraustes*	2	0	N/A
Common crossbill *Loxia curvirostra*	1	0	N/A
Linnet *Acanthis cannabina*	1	0	N/A
TOTAL	1066	45 (4%); 42	

N/A = Not applicable.

PMEs were conducted on 31 fresh and 14 frozen carcasses of finches with leg lesions. One was freshly dead, 12 were mildly autolysed, 30 were moderately decomposed and two were in a state of advanced decomposition. The state of carcass preservation and how extensive the lesions were, dictated which diagnostic tests could be performed, as summarised in Table 3. *Cnemidocoptes* sp. mites were detected in the majority (91%: 41/45 birds) of finches with leg lesions. Both microscopical examination of NaOH skin lesion digests (87%: 39/45) and histopathological examination (87%: 34/39) were similarly effective for ectoparasite detection, with high congruence between the results when both tests were conducted on the same bird: (85% (33/39) for NaOH digest and 87% (34/39) for histopathology).

**Table 3:** Diagnostic test results for *Cnemidocoptes* sp. mite detection, avian papillomavirus and avian poxvirus PCR by finch species.

Species	*Cnemidocoptes* sp. mites* % positive (no. positive/total no. tested)	Papillomavirus PCR% positive (no. positive/total no. tested)	Avian poxvirus PCR% positive (no. positive/total no. tested)	TOTAL no. of birds with leg lesions by species
Bullfinch	100% (4/4)	67% (2/3)	0% (0/3)	4
Chaffinch	90% (35/39)	73% (19/26)	0% (0/26)	39
Greenfinch	100% (1/1)	100% (1/1)	0% (0/1)	1
Goldfinch	100% (1/1)	100% (1/1)	0% (0/1)	1
TOTAL	91% (41/45)	74% (23/31)	0% (0/31)	45

Asterisk denotes the results of NaOH digest and histopathology combined.

PCR for FcPV1 was positive in samples of leg lesion from 23 of 31 (74%) birds tested: 19 chaffinch, 2 bullfinch, 1 greenfinch and 1 goldfinch. Of the four finches (all chaffinches) in which mites were not detected, samples were available for PCR testing in one case which was positive for FcPV1. A consensus sequence of 394 base pair (bp) was recovered from 20 finches. This sequence clustered with 100% sequence identity to the *BCon* region (BRD4 binding in control cells) of the FcPV1 reference genome on an NCBI Blast search (Genbank Acc. No: AY057109). A shorter consensus sequence length was obtained from three chaffinches (277–375 bp) due to poorer sequence quality: these shorter sequences obtained had 100% identity to the sequences of the other FcPV1 isolates. All sequences were submitted to Genbank (SAMN08706592- SAMN08706614; see Supplementary Worksheet S1). PCR for avian poxvirus was negative for all 31 samples tested (Table 3).

Of the 40 ‘negative control’ chaffinches which had no macroscopic leg skin lesions detected, FcPV1 was detected in five birds from which a 394 base pair PCR product identical to that derived from finches with leg lesions was generated (Genbank: SAMN08706615-SAMN08706619). Subgross examination of leg skin from the five PCR-positive ‘negative control’ chaffinches revealed equivocal evidence of mild lesions in two birds, which both had excessively flaking skin with an irregular surface.

Significant concurrent infectious disease, considered likely to be the primary cause of death, was diagnosed in 62% (28/45) of the finches found dead or euthanased with leg lesions. Concurrent disease was due to trichomonosis (based on the presence of necrotic ingluvitis lesions +/− *Trichomonas gallinae* isolation; n = 23), suspected staphylococcosis (based on isolation of *Staphylococcus aureus* from multiple sites but histopathology could not be conducted for confirmation of this diagnosis; n = 5), salmonellosis (based on *Salmonella* Typhimurium DT56v isolation from macroscopic lesions; n = 1) and suspected *Escherichia albertii*-associated disease (based on isolation from liver and small intestinal contents with 20E analytical profile index profile 4144102; n = 1). Two birds had multiple significant concurrent infections. No significant concurrent infectious disease was diagnosed in 17 finches. The cause of death in these birds was euthanasia (n = 4), blunt trauma (n = 6), predation (n = 4) and undetermined (n = 3); 13 of these birds were in normal body condition, two were thin and the body condition of two was not determined.

Tissue quality for histopathological examination varied but was considered sufficient to permit adequate interpretation of findings, for the majority (92%: 35/38) of finches. Epidermal hyperplasia was detected in all but one of the examined finches and was graded moderate or severe in 67% (26/39) of cases and mild in 31% (12/39) of cases. Examination revealed moderate to severe hyperkeratosis in the majority of finches (92%: 36/39). A mild-to-moderate degree of keratinocyte vacuolation was observed in around half the cases (18/39). Dermal inflammation was an inconsistent feature, being absent in 41% (16/39) of finches and of mild-to-moderate severity in 31% (12/39) of cases. Assessment of this histopathological feature was limited by the state of tissue preservation in 11 birds. Epidermal hyperplasia, hyperkeratosis, keratinocyte vacuolation and dermal inflammation were noted in birds with *Cnemidocoptes* sp. mites alone and in combination with FcPV1 DNA. There was poor tissue preservation of the only chaffinch that was FcPV1 PCR-positive with no mites detected, therefore assessment of histological changes typical of FcPV1 infection alone was not possible in this study. Intercellular oedema was noted in a single chaffinch which had *Cnemidocoptes* sp. mites but for which papillomavirus PCR testing was not performed. Suspected intranuclear viral inclusion bodies were observed in two chaffinches, (one of which was sequence positive for FcPV1 and one was untested). Histopathological examination found no evidence of Bollinger bodies.

**Figure 6:**
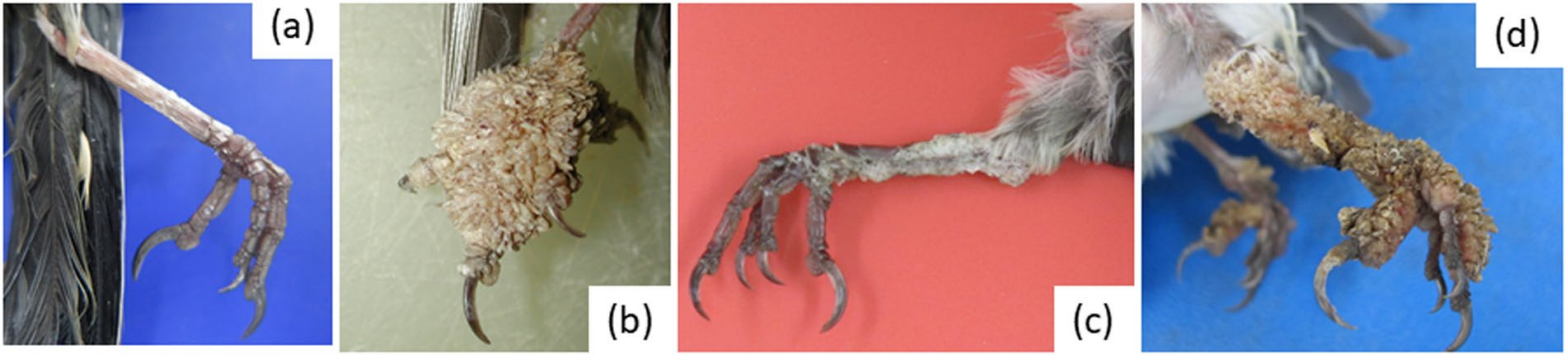
Appearance of normal chaffinch leg skin (**a**) and examples of proliferative leg skin lesions in the same host species according to three categories used in this study (**b**) papilliferous tassel-like lesions (e.g. XT0981-05) category 1 (**c**) generalised scale and/or cornified proliferative lesions with smooth surface (e.g. XT1094-13) category 2 (**d**) mixed appearance (e.g. XT507-13) category 3.

Macroscopic images of leg lesions were available from 87% (39/45) of the affected finches examined *post mortem* in the study. Examples of the range of lesion appearance are shown in Fig. 6. Lesions were of variable severity with the majority classified as severe (59%; 23/39) ranging to moderate (23%; 9/39) and mild (18%; 7/39). Both legs had lesions in the majority of finches (87%; 34/39). Missing tarsi or digits were present in a small number of finches only (13%; 5/39). Only 10% (4/39) of finches had distinct papilliferous lesions: two were both mite- and PCR-positive, samples from the other two were negative for mites and were not tested for papillomavirus. Generalised excess scale (typically white to grey-coloured) or cornified proliferative lesions with a smooth surface was present in 38% (15/39) of finches; PCR for FcPV1 was conducted in eight of these birds of which four were positive. Mites were detected in all but one of these finches. Lesions of mixed appearance, typically with tan-coloured proliferative cornified lesions with an irregular surface, some with papillary projections, were seen in the remaining 51% (20/39) of finches. PCR for FcPV1 was conducted on 17 of these birds, of which 82% (14/17) were positive. Mites were detected in all of these finches.

## Discussion

Our surveillance for leg lesions in wild finches, employing both opportunistic and systematic reporting networks, indicates that this condition is widespread in chaffinches throughout GB but occurs much less frequently in other members of the Fringillidae. Such lesions were reported from circa 4% of monitored gardens used by chaffinches during each week of our two-year period of systematic surveillance. Seasonal variation in the reporting of affected birds was greater in eastern GB, with a more pronounced peak during the winter months, than in western GB. The winter population of chaffinches in GB is substantially increased by the immigration of birds from continental Europe, predominantly from Scandinavia, with the birds typically arriving in southern and eastern England^32^. This winter arrival, along with a greater reliance on provisioned food in gardens, is likely to contribute to the observed peak of reports of affected chaffinches during the winter months. Since both FcPV1 and *Cnemidocoptes* sp. mites are believed to be transmitted by contact^1,2^, there are multiple mechanisms by which garden bird feeding stations might influence transmission, comprising direct contact between birds when they congregate at feeders and indirect transmission through contaminated feeder surfaces (e.g. perches, tables). However, it is not possible with the available data to evaluate the relative importance of risk factors for occurrence of finch leg lesions, and the extent to which supplementary feeding may alter their occurrence^33^. It is important to note that anecdotal reports are available from ringers of chaffinches with leg skin lesions, from across habitat types (RAR *pers.communication*), and that this is a gregarious species with opportunities for intraspecific contact^34^. Studies comparing the prevalence of leg skin lesions in localities of matched habitat type with and without supplementary wild bird feeding, may help address this knowledge gap^35^.

Whilst chaffinches with leg lesions have been reported in Fennoscandia in recent decades, there are few surveillance data available to quantify the prevalence of leg lesions in breeding chaffinches in continental Europe (E. Agren, K. Handeland, M. Isomursu, R. Väisänen, *pers comms*). Literák *et al*.^14^ noted chaffinches with leg lesions across seasons in the Czech Republic and Germany, but were unable to comment on whether there were temporal patterns of disease occurrence. Whilst no detailed studies that describe the rate of leg lesion progression or persistence in chaffinches are available, leg lesions associated with papillomavirus infection in captive greenfinches were observed to develop over a time course of several months^15^. The histological appearance of lesions suggested they are likely chronic, taking weeks or more to develop, so it seems likely that a proportion of continental migrants are affected by the time that they arrive in GB.

Opportunistic reports showed similar seasonality in the number of sites where finches with leg lesions were observed to the systematic surveillance. These reports had a widespread geographical distribution across GB, with greatest occurrence in the East of England, where the influence of overwintering chaffinches was predicted to be greatest. Furthermore, this seasonal increase in the eastern regions begins in October when the majority of overwintering chaffinches arrive in GB. Opportunistic surveillance reliant on *ad hoc* sightings of wildlife disease incidents is vulnerable to reporting bias, since there is no control over observer effort in time or space^36^. Also, members of the public are more likely to report sightings of wild bird mortality, particularly involving multiple individuals and are less likely to report conditions associated with morbidity (such as leg lesions) alone (BL, *personal observation*). This might explain why the dataset of opportunistic surveillance reports is relatively small with 80–90 disease sightings recorded each year. Complementary to the opportunistic reporting system, our systematic surveillance provided information from a large number of sites across GB which were monitored with a consistent level of intensity throughout each year. The results from these sites demonstrate the value of this citizen science approach for monitoring the health of garden birds with externally visible, highly characteristic clinical signs.

Of the wild finch carcasses examined from across England and Wales, leg lesions were found in 16% of chaffinches, 9% of bullfinches, 1% of goldfinches and 0.2% of greenfinches. To the best of our knowledge, proliferative leg skin lesions have not been documented previously in goldfinches. The carcasses examined *post mortem* comprised a convenience sample, a non-probability sampling technique where samples are selected because of their availability, but do not meet random sampling assumptions. The species bias likely introduced by differential species susceptibility to concurrent disease, most notably finch trichomonosis, an emerging disease causing epidemic mortality in GB since 2005, confounds the species composition of PME submissions and means that robust statistical comparison of the extent of interspecific variation of leg lesions is not possible with this dataset. Greenfinches are the species most frequently diagnosed with finch trichomonosis and are consequently the modal wild bird species examined by GWH, whilst chaffinches are the second most frequently affected species^33^.

Our results, however, do indicate that the greenfinch and goldfinch are infrequently affected with proliferative leg skin lesions, with the majority of opportunistic reports involving sightings of single or small numbers of affected chaffinches. The population of chaffinches in GB is large (circa 13 million breeding individuals)^37^. Since the mortality rates of birds affected by cnemidocoptosis and/or papillomatosis appear to be low, these diseases do not appear to pose a conservation threat but they probably adversely impact the welfare of affected birds. A 10-year study of circa 400,000 wild birds of almost 175 species caught at ringing stations in the Netherlands found evidence of leg lesions described as “papillomas” in 330 of circa 250,000 (circa 1.3%) chaffinches and an unspecified, small number of similarly affected bramblings: no leg lesions were observed in any other wild bird species^11^. Whilst circa 1 million birds are ringed annually in Great Britain and Ireland^38^, there is no process to systematically collect and record sightings of disease. Based on anecdotal reports from bird ringers in GB, however, proliferative skin lesions affecting the legs of finches occur at low prevalence (R.A.R. *personal communication*).

*Cnemidocoptes* mites and FcPV1 DNA were detected in each of the four finch species with leg lesions that were examined *post mortem*. Both of these agents are known to cause proliferative skin lesions of the legs in finches and their co-detection was confirmed in the majority of birds. As has previously been hypothesised^19^, infection with either FcPV1 or *Cnemidocoptes* sp. mites might predispose to infection with the other, e.g. via the development of proliferative lesions breaching the skin integrity.

In the majority of finches examined *post mortem*, the leg lesions were determined to be unrelated to the COD. It is possible that some of the finches with leg lesions that died of predation and/or other trauma had impaired locomotion which predisposed them to injury. Nevertheless and consistent with other studies^3,11^, all but one of the birds with no concurrent infectious disease were in normal body condition, indicating that they had continued to feed normally despite the presence of leg lesions. We note that the availability of easily accessible supplementary food at garden stations may have enabled the affected birds to maintain their body condition, which might not be the case in other habitat types where similar high energy food sources are not available. Reports from members of the public assisting with our surveillance also indicate that finches with leg lesions generally appear able to cope well, with no significant impediment to feeding or locomotion (B.L., *personal communication*). Digit loss has been described in severe cases of cnemidocoptosis and papillomatosis^1,3^ and such lesions, which might have compromised the bird’s ability to perch, were observed in a minority (13%) of affected finches examined in the current study.

The BCon region of the L1 gene, used for molecular identification of avian papillomaviruses, is relatively well conserved between papillomavirus species. FcPV1 and *Serinus canaria* papillomavirus 1, the papillomavirus most similar to FcPV1, share less than 68% nucleotide identity. In contrast, known FcPV1 sequences differ in this region of the genome by a maximum of 1 nucleotide^6,31^. In the current study, we document the first papillomavirus sequences for bullfinch, greenfinch and goldfinch. The molecular detection of a papillomavirus fragment which is 100% identical to FcPV1 in each of these three finch species is particular noteworthy given the general understanding that papillomaviruses are host-adapted and species-restricted^39^, although “Clay’s Rule” indicates that related papillomaviruses might infect related species^9^. There is some evidence that papillomaviruses are not always species-restricted. *Sylvilagus floridanus* PV1, from the western cottontail rabbit (*S. floridanus*), infects multiple species of rabbit in experimental studies^40^. Two species of papillomavirus, EserPV2 and EserPV3, originally detected in the serotine bat (*Eptesicus serotinus*) have also been detected in a different, but closely related bat species, the meridional serotine (*E. isabellinus*)^41^ and four species of bovine papillomavirus from *Bos taurus* have been found in multiple other species. These have generally been found in species belonging to the same order as cattle, Artiodactyla (bison, buffalo, giraffe, sable antelope (*Hipotrachus niger*) and yak), but also include Perissodactyla (donkey, horse, tapir and zebra) and, in the case of BPV14, one Carnivora species (*Felis catus*)^42^. This suggests host-specificity might be less strict than is currently accepted. Full characterization of the L1 protein is required for papilloma type designation.

Although we have detected FcPV1 DNA in finches, we have not confirmed this virus as the aetiology of the skin lesions seen in any of the birds examined, so it is possible its presence is an incidental finding. However, FcPV1 DNA was detected in only three (7.5%) of the unaffected ‘negative control’ birds examined, indicating that the presence of the virus in the absence of skin lesions is fairly uncommon. Unaffected PCR-positive birds might be aclinical carriers or in the early stages of disease incubation. Whilst FcPV1 DNA was detected by PCR in multiple affected finches, the test used does not discriminate active infection from latent infection or from environmental contamination; for example, from perches shared with infected finches. Latent infection can occur in other species with papillomavirus infection (e.g. regression of human papillomas can lead to maintenance of latent virus in basal cells^43^). Future development of a serological tool to detect antibodies against FcPV1 would help inform the extent of wild chaffinch exposure to FcPV1.

FcPV1 sequences (including one full genome of ca. 7700 bp) have been previously reported from chaffinches in Madeira, the Netherlands, Sweden, Spain and Italy^6,11,13,31,44^. All the sequences recovered in the current study, indeed all modern sequences, are identical, belonging to one dominant haplotype. It is highly likely that they are the same papillomavirus, FcPV1^44^ and there is no currently published evidence that other strains circulate in chaffinch. Comparison of full finch papillomavirus genomes would determine if the full viral genome is as conserved as the gene fragment we sequenced, whether variation in the circulating strains occurs across the species range and whether variation in the viral strain exists between avian host species.

Hyperkeratosis, hyperplasia and epidermal papillary projections were common histopathological features of finch leg lesions but none of these proved useful for discriminating between the relative importance of *Cnemidocoptes* sp. mite infestation as compared with FcPV1 infection in lesion development. Detection of *Cnemidocoptes* sp. mites and subsequent diagnosis of cnemidocoptosis was straightforward using a NaOH digest or histopathological examination, since - when this disease is present - mite infestation can easily be co-localised with tissue reaction. Histopathological examination provided equivocal evidence of intranuclear inclusion bodies, as has been previously described in papillomatosis^16^, in only two chaffinches: one had leg lesions classified as papilliferous based on macroscopic appearance and had no evidence of mite infestation, but no FcPV1 PCR was conducted; the second had leg lesions classified as mixed macroscopic appearance and was FcPV1 PCR-positive with concurrent mite infection. Co-localisation of virus and lesion is required to elucidate the significance of FcPV1 DNA detection. Whilst Jacobson *et al*.^45^ detected FcPV1 using immunohistochemistry, no papillomavirus-specific antibodies were available for the current study and attempts to develop *in-situ* hybridisation (ISH) using biotin-labelled probes were unsuccessful (APHA, *unpublished data*), perhaps as a consequence of long-term tissue storage in formalin prior to ISH processing. Electron microscopy to detect papillomavirus virions, as has been used in other studies (e.g. Prosperi *et al*.^6^), offers an alternative for confirmation of viral infection; however, such studies require optimal tissue preservation which was not possible in the current study due to the nature of sample acquisition.

On blind review of the macroscopic lesions of the birds examined *post mortem*, we found that, due to the high prevalence of both *Cnemidocoptes* sp. mites and FcPV1 DNA and a range of abnormalities detected from mild generalised scale to severe papilliferous lesions, it was not possible to confidently discriminate birds infected with the two agents, or to identify birds with likely co-infection. When Literak *et al*.^19^ described co-infection in a chaffinch with leg lesions, they noted that co-infection could not be determined from lesion appearance and they highlighted the importance of laboratory examinations for the detection of papillomavirus, *Cnemidocoptes* mites and avipox virus. This study supports the hypothesis proposed by Literak *et al*.^19^ that co-infection with *Cnemidocoptes* sp. and FcPV1 in finches with leg lesions is more frequent than previously considered. Investigation of the possible interplay of these pathogens in the pathogenesis of these lesions is required.

## Electronic supplementary material

Supplementary files

Supplementary Worksheet

Supplementary Materials
