# Supplementary material for: Spatio-temporal dynamics and aetiology of proliferative leg skin lesions in wild British finches
Source: Sci Rep. 2018 Oct 10;8:14670. doi: 10.1038/s41598-018-32255-y (PMC6180014; doi:10.1038/s41598-018-32255-y)
Supplement: Supplementary file 1 — Supplementary files [file 41598_2018_32255_MOESM1_ESM.docx]

**Spatio-temporal dynamics and aetiology of proliferative leg skin lesions in wild British finches**

Becki Lawson^1*^, Robert A. Robinson^2,^ Julia Rodriguez-Ramos Fernandez^3^, Shinto K. John^1^, Laura Benitez^4^, Conny Tolf^5,^ , Kate Risely^2^, Mike P. Toms^2,^ Andrew A. Cunningham^1^, Richard A. J. Williams^5,6^

**SUPPLEMENTARY MATERIALS**

**Supplementary Table S1:** Parameter estimates from a mixed model in which proportion of gardens with chaffinches that also reported individuals with leg lesions was modelled as a function of time of year (Week No), number of chaffinches reported per garden (N Chaff) and GOR (see text). In the lower part of the table the estimated random effects are given on the response scale (i.e. % gardens reporting lesions) for the intercept and the logit-predictor scale for the slopes for each level.

| Fixed Effects |  | Random Effects |  |  |  |
| --- | --- | --- | --- | --- | --- |

| Intercept | | β = -3.91 ± 0.28 | |  | σ^2^ = 0.90 |  |  |  |  |
| --- | --- | --- | --- | --- | --- | --- | --- | --- | --- |
| N Chaffs | | β = 0.079 ± 0.043 | |  | σ^2^ = 0.10 |  |  |  |  |
| s(Week No) | | edf = 6.26 | |  |  |  |  |  |  |
| Residual | |  | |  | σ^2^ = 0.94 |  |  |  |  |
| Intercept Estimates | | | |  |  |  |  |  |  |
| East | E Mids | | E Engl | | London | NE Engl | SE Engl | S Scot | Yorks |
|  | 0.018 | | 0.023 | | 0.011 | 0.006 | 0.037 | 0.004 | 0.013 |
| West | N Scot | | NW Engl | | SW Engl | Wales | W Mids |  |  |
|  | 0.008 | | 0.058 | | 0.044 | 0.036 | 0.067 |  |  |
| Slope Estimates | | |  | |  |  |  |  |  |
| East | E Mids | | E Engl | | London | NE Engl | SE Engl | S Scot | Yorks |
|  | 0.16 | | 0.15 | | 0.15 | 0.15 | 0.15 | 0.13 | 0.14 |
| West | N Scot | | NW Engl | | SW Engl | Wales | W Mids |  |  |
|  | 0.025 | | -0.079 | | 0.061 | 0.027 | -0.085 |  |  |

Alternative table not excluding the three points

| Fixed Effects | | | |  | Random Effects | |  |  |  |
| --- | --- | --- | --- | --- | --- | --- | --- | --- | --- |
| Intercept | | β = -3.78 ± 0.28 | |  | σ^2^ = 0.89 |  |  |  |  |
| N Chaffs | | β = 0.081 ± 0.043 | |  | σ^2^ = 0.079 |  |  |  |  |
| s(Week No) | | edf = 6.41 | |  |  |  |  |  |  |
| Residual | |  | |  | σ^2^ = 0.94 |  |  |  |  |
| Intercept Estimates | | | |  |  |  |  |  |  |
| East | E Mids | | E Engl | | London | NE Engl | SE Engl | S Scot | Yorks |
|  | 0.024 | | 0.026 | | 0.013 | 0.007 | 0.047 | 0.005 | 0.015 |
| West | N Scot | | NW Engl | | SW Engl | Wales | W Mids |  |  |
|  | 0.008 | | 0.060 | | 0.050 | 0.040 | 0.064 |  |  |
| Slope Estimates | | |  | |  |  |  |  |  |
| East | E Mids | | E Engl | | London | NE Engl | SE Engl | S Scot | Yorks |
|  | 0.068 | | 0.094 | | 0.090 | 0.110 | 0.063 | 0.115 | 0.089 |
| West | N Scot | | NW Engl | | SW Engl | Wales | W Mids |  |  |
|  | 0.022 | | -0.096 | | 0.023 | 0.005 | -0.073 |  |  |

**Supplementary Figure S1a-b**: **Regional occurrence of chaffinch leg lesion reports in (a) 2014 and (b) 2015 reported by opportunistic surveillance**. Shading indicates the number of gardens reporting diseased individuals through the Garden Wildlife Health website ([www.gardenwildlifehealth.org](http://www.gardenwildlifehealth.org)) per 100,000 gardens in the region. Map was created using ArcMap 10.0 (<https://desktop.arcgis.com/en/arcmap/)>.

| **(a)** | **(b)** |
| --- | --- |
| 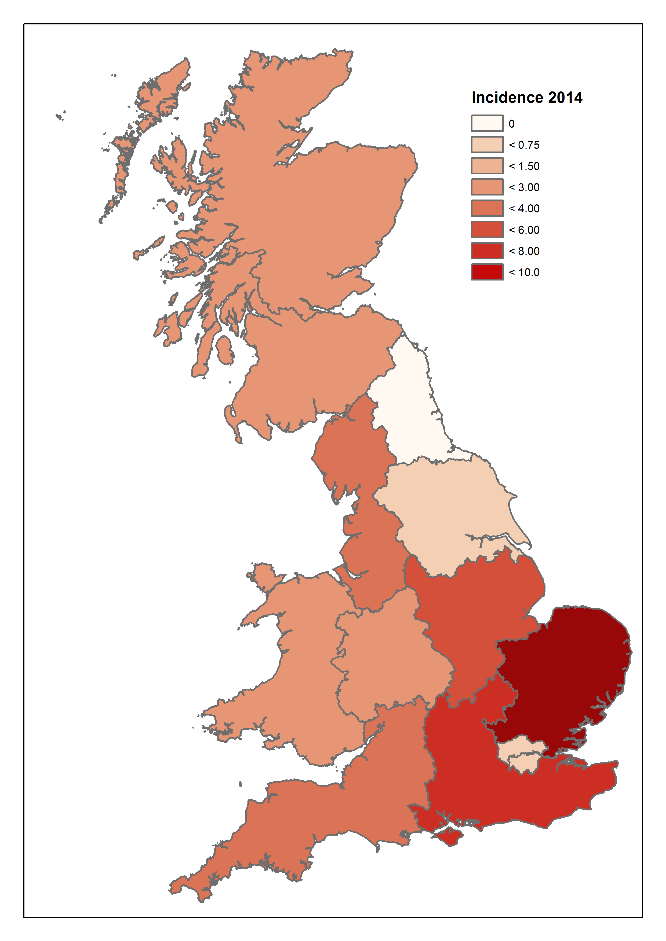 | 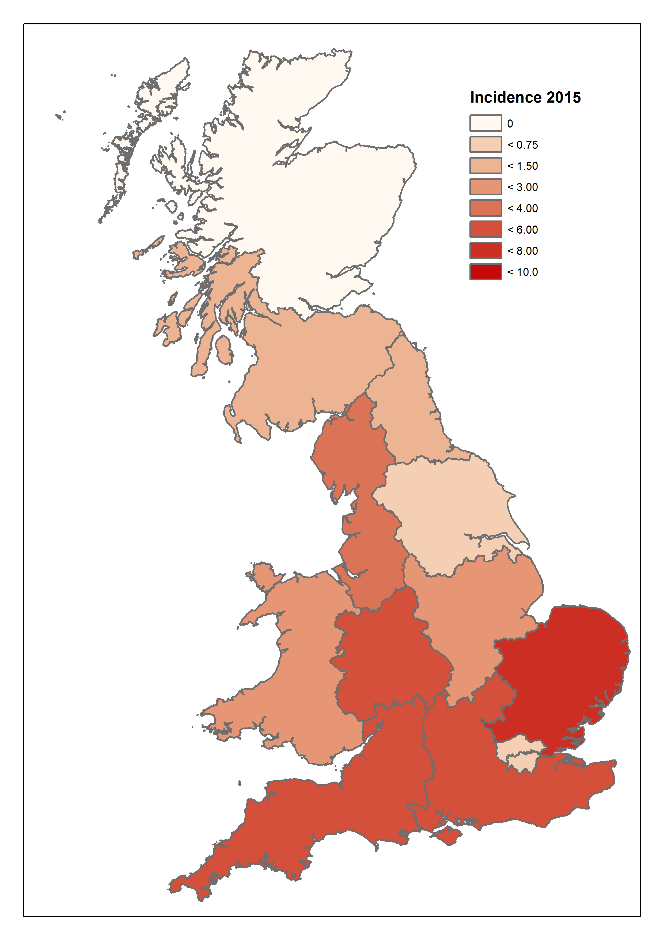 |

**Supplementary Figure S2**: Mean proportion of gardens surveyed as part of the BTO’s Garden BirdWatch scheme in which chaffinches were reported in each region.

●

●

●

●

●

●

●

●

●

●

●

●

0.50

0.55

0.60

0.65

0.70

0.75

0.80

SW Eng.

W Mids

Wales

NW Eng.

N Scot.

S Scot.

NE Eng.

Yorks.

E Mids

E. Eng.

London

SE Eng.
