# Supplementary material for: Spatio-temporal dynamics and aetiology of proliferative leg skin lesions in wild British finches
Source: Sci Rep. 2018 Oct 10;8:14670. doi: 10.1038/s41598-018-32255-y (PMC6180014; doi:10.1038/s41598-018-32255-y)

Spatio-temporal dynamics and aetiology of proliferative leg skin lesions in wild British finches

Becki Lawson[1*], Robert A. Robinson[2,] Julia Rodriguez-Ramos Fernandez[3], Shinto K. John[1], Laura Benitez[4], Conny Tolf[5,] , Kate Risely[2], Mike P. Toms[2,] Andrew A. Cunningham[1], Richard A. J. Williams[5,6]

SUPPLEMENTARY MATERIALS

Supplementary Table S1: Parameter estimates from a mixed model in which proportion of gardens with chaffinches that also reported individuals with leg lesions was modelled as a function of time of year (Week No), number of chaffinches reported per garden (N Chaff) and GOR (see text). In the lower part of the table the estimated random effects are given on the response scale (i.e. % gardens reporting lesions) for the intercept and the logit-predictor scale for the slopes for each level.

Fixed Effects			Random Effects				
Intercept	$\beta = -3.91 \pm 0.28$		$\sigma^2 = 0.90$				
N Chaffs	$\beta = 0.079 \pm 0.043$		$\sigma^2 = 0.10$				
s(Week No)	edf = 6.26						
Residual			$\sigma^2 = 0.94$				
Intercept Estimates							
East	E Mids	E Engl	London	NE Engl	SE Engl	S Scot	Yorks
	0.018	0.023	0.011	0.006	0.037	0.004	0.013
West	N Scot	NW Engl	SW Engl	Wales	W Mids		
	0.008	0.058	0.044	0.036	0.067		
Slope Estimates							
East	E Mids	E Engl	London	NE Engl	SE Engl	S Scot	Yorks
	0.16	0.15	0.15	0.15	0.15	0.13	0.14
West	N Scot	NW Engl	SW Engl	Wales	W Mids		
	0.025	-0.079	0.061	0.027	-0.085		

Alternative table not excluding the three points

Fixed Effects			Random Effects				
Intercept	$\beta = -3.78 \pm 0.28$		$\sigma^2 = 0.89$				
N Chaffs	$\beta = 0.081 \pm 0.043$		$\sigma^2 = 0.079$				
s(Week No)	edf = 6.41						
Residual			$\sigma^2 = 0.94$				
Intercept Estimates							
East	E Mids	E Engl	London	NE Engl	SE Engl	S Scot	Yorks
	0.024	0.026	0.013	0.007	0.047	0.005	0.015
West	N Scot	NW Engl	SW Engl	Wales	W Mids		
	0.008	0.060	0.050	0.040	0.064		
Slope Estimates							
East	E Mids	E Engl	London	NE Engl	SE Engl	S Scot	Yorks
	0.068	0.094	0.090	0.110	0.063	0.115	0.089
West	N Scot	NW Engl	SW Engl	Wales	W Mids		
	0.022	-0.096	0.023	0.005	-0.073		

Supplementary Figure S1a-b: **Regional occurrence of chaffinch leg lesion reports in (a) 2014 and (b) 2015 reported by opportunistic surveillance**. Shading indicates the number of gardens reporting diseased individuals through the Garden Wildlife Health website (www.gardenwildlifehealth.org) per 100,000 gardens in the region. Map was created using ArcMap 10.0 (https://desktop.arcgis.com/en/arcmap/).

(a) **(b)**

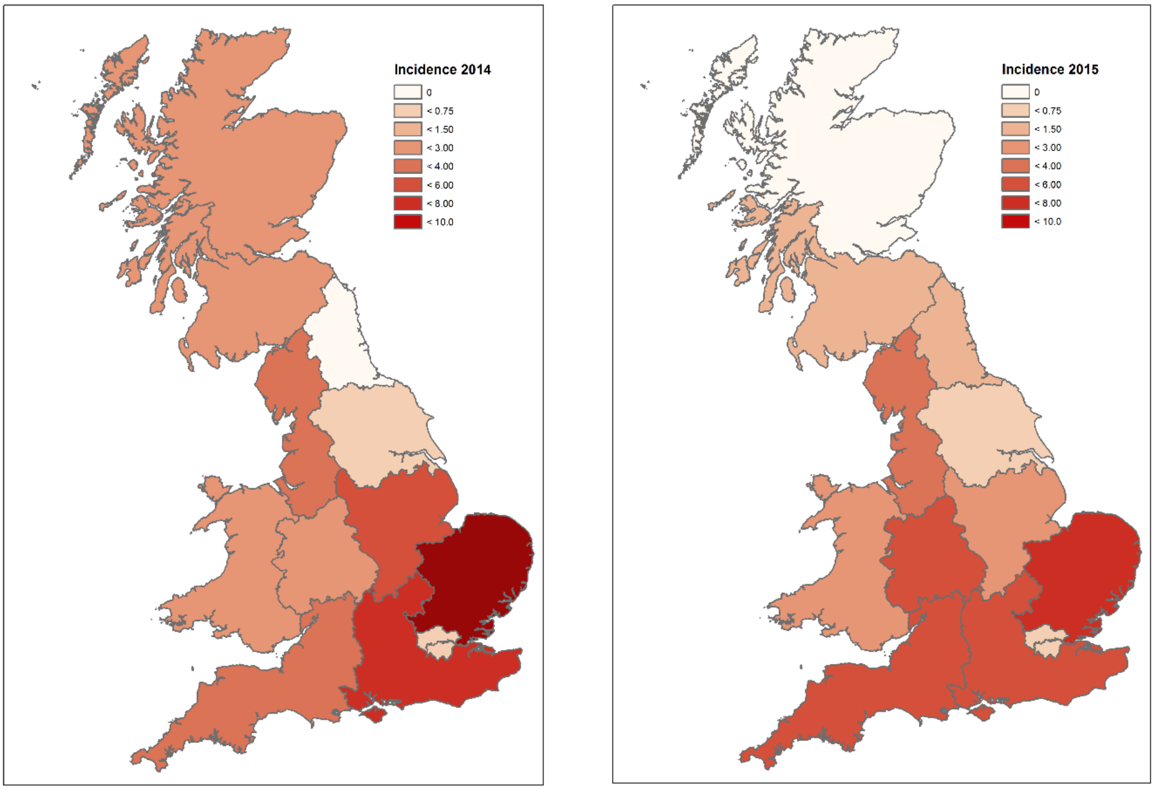

Supplementary Figure S2: Mean proportion of gardens surveyed as part of the BTO's Garden BirdWatch scheme in which chaffinches were reported in each region.

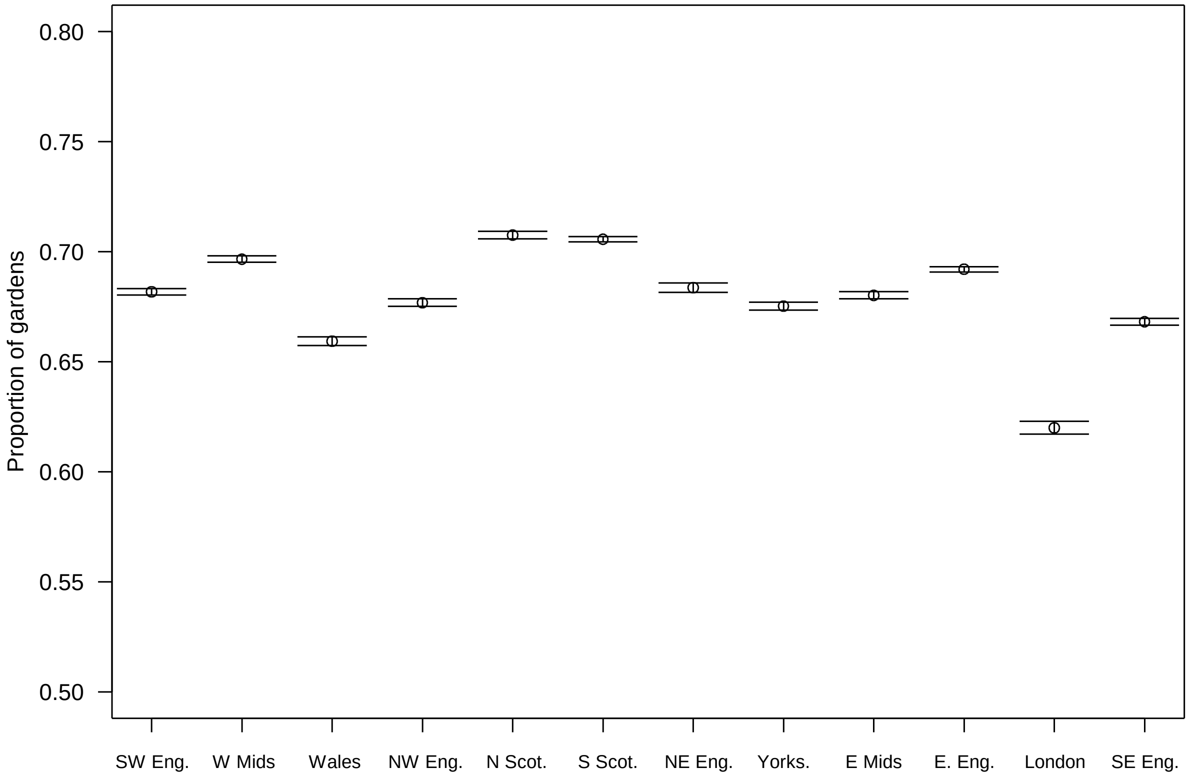

Supplement: Supplementary file 3 — Supplementary Materials [file 41598_2018_32255_MOESM3_ESM.pdf]
